# Nest attributes influence choice accuracy, but not decision latency in acorn ants

**DOI:** 10.1371/journal.pone.0329528

**Published:** 2026-01-16

**Authors:** Sheila Shu-Laam Chan, Isaac P. Weinberg, Philip T. Starks, Claire T. Hemingway

**Affiliations:** 1 Department of Biology, Tufts University, United States of America; 2 Department of Ecology and Evolutionary Biology, University of Tennessee, Knoxville, United States of America; 3 Collaborative for Animal Behavior, University of Tennessee, Knoxville, United States of America; 4 Department of Psychology and Neuroscience, University of Tennessee, Knoxville, United States of America; University of Vienna: Universitat Wien, AUSTRIA

## Abstract

Decision making can have significant fitness consequences across various aspects of animal life. For acorn ants, *Temnothorax curvispinosus*, choosing a new nest quickly and accurately can affect the survival and fitness of the whole colony. When emigrating, ants consider several nest attributes such as cavity shape, height, and brightness. Ants may benefit from having more attributes differentiating potential nests only if they can quickly and accurately assess all possible attributes and make well-informed decisions. Here, we asked if the number and type of attributes differentiating potential nests affected the accuracy and latency of colony decision-making. We used pair-wise tests, where potential nests differed in one to three attributes, with one nest within the pair considered less optimal. We recorded which nest colonies chose and the time it took to make the decision. We found that accuracy increased with the number of attributes, particularly when nest brightness was manipulated, indicating that increasing the number of attributes may help facilitate nest-site selection. We also found that the degree of difference did not affect the decision-making latency, suggesting that ant colonies searching for a new nest might be constrained temporally when selecting a new nest site.

## Introduction

Animals routinely make decisions with fitness consequences, such as selecting a mate or deciding what to eat or where to live [1,2]. Choices across a variety of contexts are often characterized by multiple attributes. For instance, when selecting a flower to visit, pollinators attend to multiple aspects of nectar rewards, including concentration, volume, variance, and handling time [3,4]. In most cases, these attributes are informative about different properties of choices, and attending to them may lead to better decision outcomes [5,6]. However, access to more information about potential options will only benefit decision-makers if they can quickly and accurately identify the various attributes of available options and make appropriate decisions using that information.

These complex decision scenarios are particularly common in acorn ants (*Temnothorax sp.*), which live in cavities like fallen acorns or hickory nuts, primarily on the forest floor. Given the relative instability of these nesting sites, acorn ants quickly respond to changing conditions, relocating to a new nest when their current one becomes unsuitable [7,8]. During emigration, ants likely have several potential nests to choose from, as multiple acorns have dropped from a single tree and been hollowed out by other insects at any given time. When confronted with multiple nests, acorn ants send scouts to carefully assess these options by examining several physical nest attributes, including brightness, cavity shape and size, and entrance width. This complex nest-selection process requires ants to integrate information within and across potential nest sites. Scouts then recruit more ants to preferred nest sites via tandem running [9]. When the number of ants present at a potential nest site exceeds a quorum threshold, the recruiters generally switch from tandem running to carrying of the remaining colony members, including the brood and queen(s). As the number of ants in the new nest increases, the rate of the emigration process also increases (i.e., the ants collectively decide on a new nest via positive feedback) [8]. During this process, the quality of the candidate nest affects how quickly the ants decide to initiate recruitment, with better nests typically eliciting faster recruitment and migration [7,10].

Ants may benefit from having more distinguishing attributes among potential nests only if they can efficiently identify available nest sites, evaluate the attributes of each, and make well-informed decisions based on this information. Increasing variation across multiple attributes of nests also has the potential to make decision-making difficult in several ways. First, the more attributes a nest has, the more information ants must process [5,6]. For humans, choosing based on a single attribute, like color, is often easier than evaluating options that differ in color, shape, and size [11]. Second, decisions become more complex when attributes conflict and no single option is considered the best [12,13]. In ants, choosing a nest based on a good value for one attribute may result in a bad value for another. Third, not all attributes may be equally important. Previous work has shown that ants evaluate and prioritize attributes differently: brightness appears most important, followed by the height of the cavity and, finally, the entrance width [14]. Ants also learn to rely on more informative attributes that reliably predict nest quality [15]. For instance, in an experimental setting, when only one attribute differentiated options, ant colonies increased their reliance on this attribute, relative to others [15].

When multiple attributes characterize nests, this may have important consequences for decision accuracy and latency. Accuracy may improve when additional attributes increase a nest’s relative value [16]. However, in complex choice environments, individuals often rely on decision shortcuts, only considering some available information, which may result in biased or context-dependent choices [6,13,17]. Individual crevice-nesting ants make context-dependent decisions when making multi-attribute decisions with two conflicting nest attributes [18]. Similarly, decision latency can decrease as additional attributes make one option easier to choose [16] or increase because processing more information takes longer. Some decision models predict longer decision times as more information is required to reach a threshold [5,6]. Most studies of nest-site selection focus on choice accuracy, and decision latency may be an equally important outcome to consider when evaluating decision-making outcomes, especially with multi-attribute choices. Thus far, investigations into decision latency have been limited to scenarios where only one attribute – brightness – varied between candidate nests [10,19]. Individual ants took longer to decide on a new nest than colonies. Both latency and accuracy are critical components of decision-making, with significant implications for fitness, and animals often face a tradeoff between these two factors [20].

Here, we explored how the number and type of differentiating attributes influence choice accuracy and latency. We gave ant colonies two-choice tests in which potential nest sites differed in either one, two, or three attributes, and we varied the type of attributes that differentiated potential nest sites. We measured which nest the colony chose and how long it took to make the decision. We considered several hypotheses about how manipulating attribute number and type may influence choice accuracy and latency. One possibility is that ants will perform better with access to more information about nest attributes, facilitating quick and accurate decisions. Conversely, increasing the information that ants must attend to may increase the time and processing necessary to identify the preferred nest site, leading to longer and less accurate nest selection. It is also possible that accuracy and speed may trade off [20,21], such that with more information, ants may either make faster but less accurate decisions or make slower but more accurate decisions. Finally, if ants attend primarily to one attribute more than others, we may see similar patterns of decision latency and accuracy, regardless of the number of other attributes distinguishing potential nest sites.

## Methods

### Study system

Acorn ants (*Temnothorax curvispinosus*) are commonly found in eastern North American forests. An average colony of acorn ants consists of around 80 workers, but can range from 10 to 350 individuals [22]. As their common name suggests, they are cavity-dwelling ants living in fallen acorns or hickory nuts on the forest floor. We collected 10 *T. curvispinosus* colonies, which ranged in colony size from 14 to 35 workers with brood and queens. These colonies were on the smaller end of what occurs naturally, likely reflecting a sampling bias based on what we were able to find. Colonies were collected during the summer of 2021 in Middlesex Fells Reservation in Eastern Massachusetts and maintained in the lab at 21 ºC and ambient building humidity for nine months during experiments. Using a standard protocol, we provided artificial nests (50 mm × 20 mm × 20 mm) with a glass cover, a metal screen base, and wood walls as their initial nests (e.g., [14]) before they moved into home nests used for experiments. While in captivity, colonies were fed with water, a 1:1 water-sucrose solution, and mealworms, which were replenished once a week.

### Experimental overview

**Fig 1:**
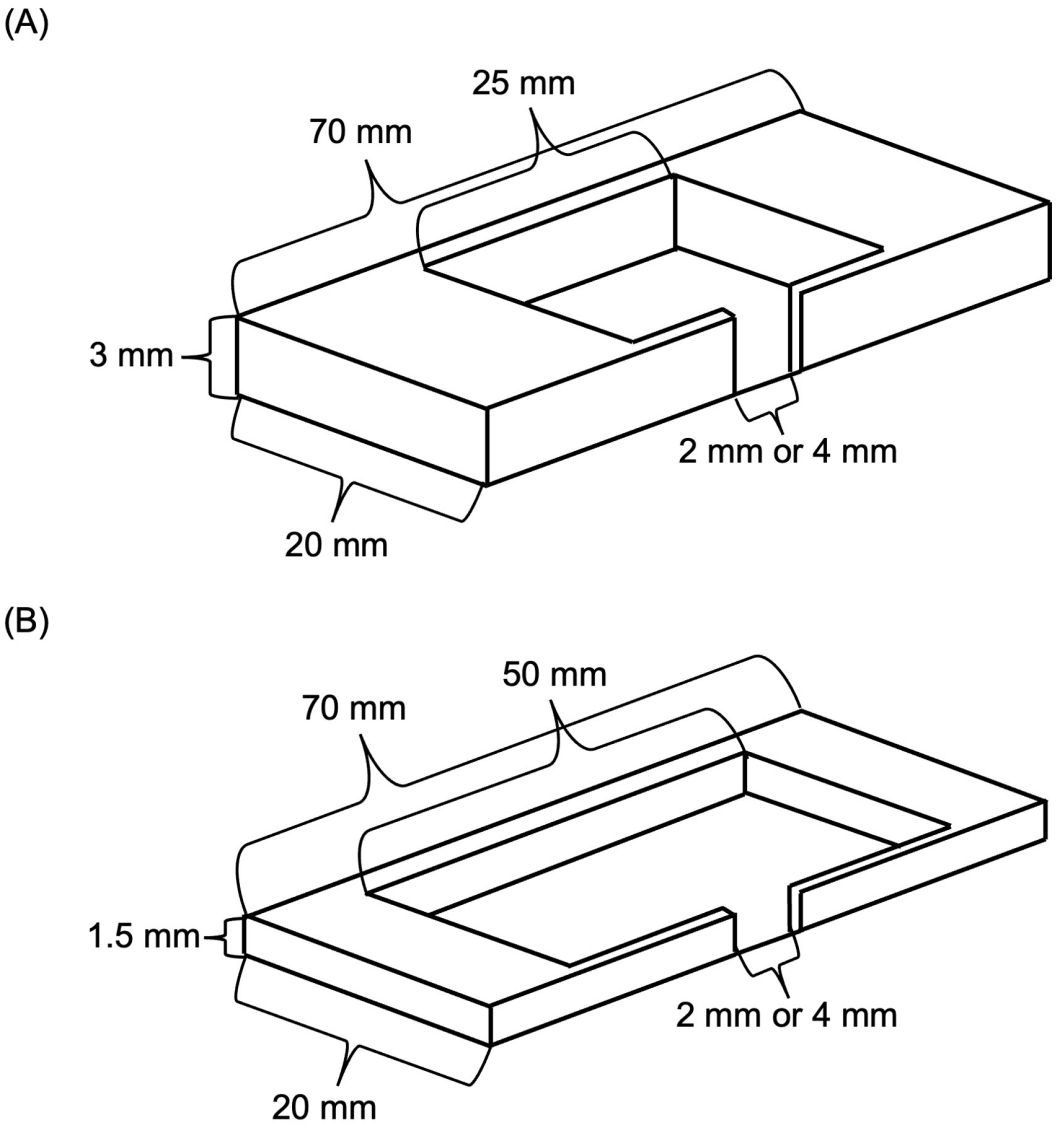
Illustration of nests used in the study. **(A)** A tall nest cavity was 25 mm × 20 mm × 3 mm, and **(B)** a short nest cavity was 50 mm × 20 mm × 1.5 mm. The dimensions of tall and short nests are different to control for the volume of nests. A narrow opening was 2 mm, and a wide opening was 4 mm. A dark nest was covered by a piece of wood and a glass microscope slide, and a bright nest had a glass slide only. The outer dimensions of all nests were 70 mm × 20 mm × 3 mm, except for bright nests, which did not have a wood plate on top.

The experiments were carried out from February 2022 to April 2022. We manipulated three nest attributes in this study: 1) nest brightness (bright or dark), 2) internal height of the cavity (tall or short), and 3) width of the nest entrance (narrow or wide). Dark nests were covered by a piece of wood to block all light. Bright nests were covered with a transparent microscope slide and exposed to an LED lamp that did not generate an observable amount of heat within the testing arena. The short and tall nest cavities were 1.5 mm and 3 mm in height, respectively. A narrow entrance was 2 mm, and a wide entrance was 4 mm (Fig 1; S1 Fig). Because of the different heights of the nests, the area of the entrance is different in the nests. The dimensions were determined based on previous experiments (S1 Table). All nests were handmade with wood and a glass cover and had approximately similar volumes (Fig 1). Previous studies have used wood as the nest material [10,23], and even though we did not test for volatile compounds, all colonies were exposed to the same material and would therefore be equally affected by any such effects. Additional wood plates were used to fill up the space outside the cavity in order to keep the outer dimension of the nests standardized (S1 Fig).

In each trial, colonies were presented with two candidate nests, which differed in the number and types of attributes, as illustrated in Table 1. Previous studies have shown that the ants prefer nests that are dark, tall, and with a narrow entrance (e.g., [14]). Six pairs of possible variable combinations were tested (n = 10) (Table 1). In Tests 1 and 2, we manipulated only one attribute to evaluate the effects of cavity *height* and entrance *width* in isolation. Tests 3 and 4 involved manipulating two nest attributes simultaneously. In Test 3, both *brightness* and *height* were manipulated to change in the same direction (i.e., in agreement). Nest 1 (target) was dark and tall, both preferred attributes, while Nest 2 (alternative) was bright and short. In Test 4, both *height* and *width* were manipulated and in conflict. Additionally, Test 4 forced the ants to compare two lower-valued attributes by eliminating the attribute that was expected to be prioritized (brightness). In Tests 5 and 6, all three nest attributes were manipulated, and both manipulations were conducted such that there was no single ‘best’ nest option, but the nest attributes were always in conflict. In Test 5, Nest 1 (target) was better than Nest 2 (alternative) in *brightness* but less attractive in the other two attributes, with a short cavity and a wide entrance. This allowed us to test whether the brightness outweighs the other two attributes when nests vary along all three. In Test 6, Nest 1 (target) was better than Nest 2 (alternative) in *brightness* and *width* but had a short cavity. This test acted as a more cognitively challenging version of Test 3, in which each nest choice had various attributes in conflict.

**Table 1:** Nest pairs tested. In all tests, Nest 1 (target) was considered better than Nest 2 (alternative) based on findings from previous studies. The differences between nests are bolded. All tests were done with 10 colonies.

Test No.	No. of differences	Nest 1 (target)	Nest 2 (alternative)
1	1	Dark, short, & **narrow**	Dark, short, & **wide**
2	1	Dark, **tall,** & wide	Dark, **short**, & wide
3	2	**Dark**, **tall**, & wide	**Bright**, **short**, & wide
4	2	Bright, **tall**, & **wide**	Bright, **short**, & **narrow**
5	3	**Dark**, **short**, & **wide**	**Bright**, **tall**, & **narrow**
6	3	**Dark**, **short**, & **narrow**	**Bright**, **tall**, & **wide**

### Experimental setup

**Fig 2:**
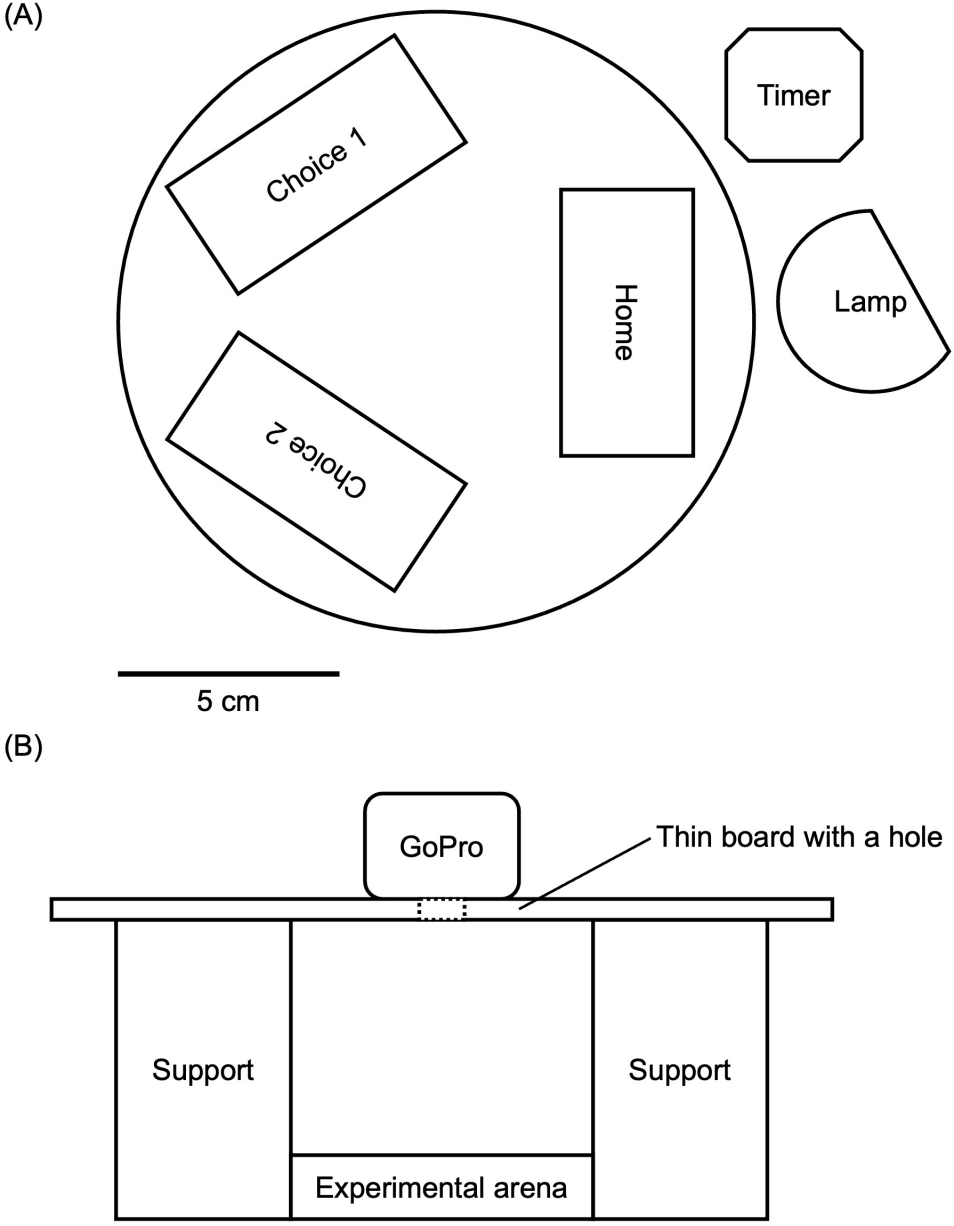
Set-ups of (A) experimental arena and (B) filming equipment (side view). Two candidate nests were placed approximately 70 mm from the home nest. The entrances of the nests all faced the center of the experimental arena. The lens of the GoPro faced down to film the arena from a top-down angle.

The experimental arena consisted of two candidate nests, placed at an equal distance (approximately 70 mm) from the home nest, within a covered petri dish (Fig 2A). We placed a timer next to the nests to measure the trial’s duration. To stimulate the initial emigration, we removed the cover from the home nest and placed an LED lamp 10 cm away to create a bright environment. We recorded emigration events using a GoPro camera, which filmed the trial in time-lapse (pictures taken at 2-s intervals) from a top-down angle (Fig 2B).

### Experimental protocol

Each colony (n = 10) encountered all six choice tests (Table 1). We randomized the order of presentation within and across colonies to minimize effects of learning or experience on emigration behavior [24,25]. Prior to testing, colonies were housed in a home nest with the best possible attributes (i.e., dark, tall, and with a narrow entrance). To initiate a trial, the roof of the home nest was removed to simulate a destroyed home nest and encourage emigration. Following nest destruction, ants would move quickly within the nest but not leave immediately. After about a minute, one to two scouts would typically leave and begin exploring the arena. A trial lasted until all the ants and brood were moved into a chosen nest, or the colony did not actively search for a new nest or initiate emigration after 1 hour. Colonies were classified as inactive if no scouts were sent out by this point, as it seemed unlikely that scouting activity would begin even with additional time. We note that this classification is based on the assumption that scouting was unlikely to begin even with additional time, although a single scout emerging later could theoretically initiate an emigration. In our experiment, both options needed to be sampled during the scouting period for a colony’s behavior to count as a choice. If a colony temporarily split and later reunited during the emigration phase, this was also recorded as a choice. We observed two such split-and-reunite decisions made by the same colony in Tests 1 and 3. However, if a colony split and did not reunite, we recorded this as no choice. This occurred once, in Test 6. Across all trials, we recorded 1) the nest selected, 2) the duration between the first scout, which is the first ant entering the arena following destruction of the home nest, leaving the home nest and the beginning of carrying (decision made), and 3) the duration between the first scout leaving the home nest and the end of the emigration event (decision finalized). While colony emigration is the point at which a decision is conclusively complete, we included both latency measures as colonies varied in size and in their number of brood, both of which may influence emigration time, regardless of nest preferences (but see [26,27]). Following a trial, each colony was placed back into the storage box with its restored home nest to allow the colony to return to its home nest before being retested. Intervals between trials were 4–7 days. If the colony decided on and moved into a new nest, this nest was put into the storage box together with the home nest. If the chosen nest was dark, the wood cover, but not the glass slide, would be removed so the colony would re-emigrate into their home nest; if it was bright, the colony would re-emigrate into the dark home nest once they found it without the chosen nest being destroyed. It was important for this experiment that colonies always started from the same nest type, as ants may incorporate experience with previous nests when evaluating new nest sites [25].

### Data analysis

Analyses were carried out using R v. 4.3.1 [28]. We used generalized linear models (GLMs) and mixed models (GLMMs) with the glm() and glmer() functions in the ‘lme4’ package [29]. We first addressed whether nest preferences varied according to the number of attributes differentiating potential nest sites or whether preferences were variable between the test conditions, depending on the types of attributes manipulated. To test for the effects of number and type of attributes on nest preferences, we ran binomial GLMs (link = logit) with nest type (target or alternative) as the response variable and either ‘attribute number’ or ‘test type’ as predictor variables. We did not include ‘colony’ as a random factor due to a singularity issue, but we included it instead as a fixed effect in all models. We also tested for effects of ‘trial order’ for each nest comparison and ‘colony size’ as potential covariates in our models. These were removed if they did not significantly improve model fit. We used the Anova() function in the ‘car’ package [30] to evaluate model fit by generating Wald chi-square tests for categorical fixed effects. Where we found significant effects (p < 0.05) of a predictor variable, we used the function and package ‘emmeans’ for *post hoc* comparisons (Tukey’s honestly significant difference HSD tests) [31]. We used the simulateResiduals() and testOverdispersion() functions in the ‘DHARMa’ package [32] to test for overdispersion, and have checked the model assumptions for the models.

To determine whether the number or type of attributes influenced either measure of decision latency, we ran GLMMs, now with a gamma distribution (link = log), commonly used for duration data with a positive right skew. We again included either ‘attribute number’ or ‘test type’ as the predictor variable. Here, we included ‘colony’ as a random factor. Similarly, we tested for effects of both ‘order’ and ‘size’ as covariates in our models and removed them when insignificant. We again used the Anova() function in the ‘car’ package [30] to evaluate model fit, and we used the function and package ‘emmeans’ for *post hoc* comparisons (Tukey’s honestly significant difference HSD tests) [31].

We also ran an additional analysis to see if the probability of colonies emigrating (S2 Table) differed among treatments. We ran GLMMs with a binomial distribution and tested whether the colonies emigrated as our response variable. Similar to the models above, we included either ‘attribute number’ or ‘test type’ as the predictor variable and ‘colony’ as a random factor. The effects of ‘order’ and ‘size’ were also tested and removed if insignificant. The Anova() function in the ‘car’ package [30] and the function and package ‘emmeans’ for *post hoc* comparisons (Tukey’s honestly significant difference HSD tests) [31] were used.

## Results

### Choice accuracy

**Fig 3:**
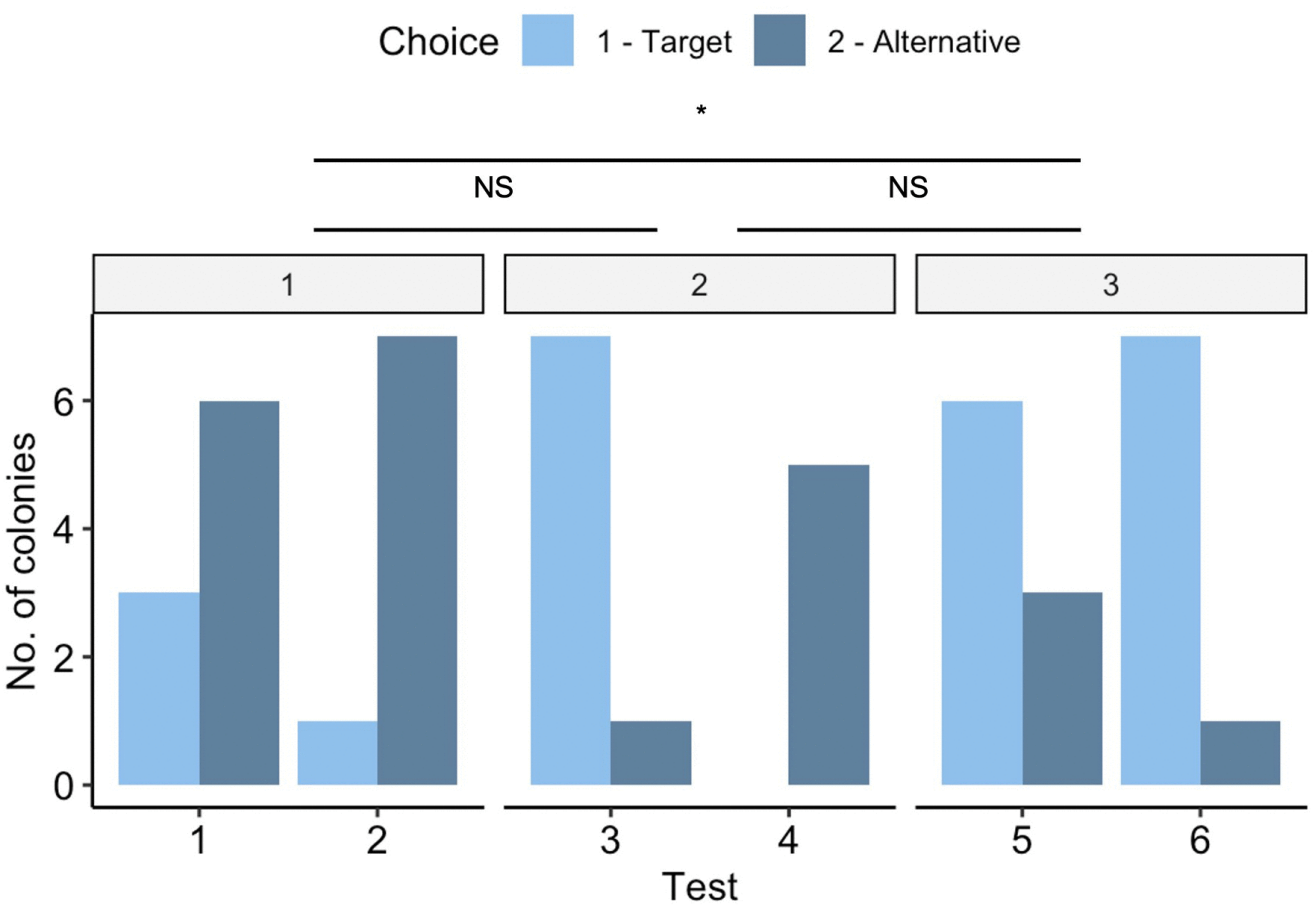
Choice accuracy across test types and the number of differences. Choices between potential nests depended on the number of differences between nest pairs and the six test types. The attribute number is denoted at the top of the figure in the grey box, and the test type is plotted along the x-axis. The target nest was designed to be more attractive than the alternative nest. The number of differences and the test type significantly affected which nest the colonies chose.

Increasing the number of attributes that differentiated potential nest sites significantly increased the probability that the target nest would be selected over the alternative (χ^2^ = 9.918, df = 2, p = 0.007; Fig 3). Increasing from one to two attributes did not significantly increase choice accuracy for the target nest across the choice tests (z = − 1.609, p = 0.242), nor did increasing from two to three (z = −1.321, p = 0.383). However, nest accuracy significantly increased with three attributes relative to one attribute (z = −2.894, p = 0.011), even though the three attributes here were never in agreement. Colony ID had no effect on accuracy (χ^2^ = 0.739, df = 1, p = 0.390). There was also no effect of order (χ^2^ = 0.312, df = 5, p = 0.577; S2 Fig) or colony size (χ^2^ = 0.005, df = 1, p = 0.945) on choice accuracy, so they were removed from the final model.

Choice accuracy differed significantly across the six test types (χ^2^ = 24.269, df = 5, p < 0.001; Fig 3), but this was not significantly different in *post hoc* comparisons. In Test 2, these results were in the opposite direction from what was expected, with ants preferring the nest with the shorter cavity over the taller. In Tests 3 and 6, preferences were almost exclusively for the darker nest, regardless of other attribute values. Colony ID had no effect (χ^2^ = 1.045, df = 1, p = 0.307). There was also no effect of order (χ^2^ = 0.016, df = 1, p = 0.900; S2 Fig) or colony size (χ^2^ = 0.009, df = 1, p = 0.925) on choice accuracy, so they were removed from the final model.

### Choice latency

**Fig 4:**
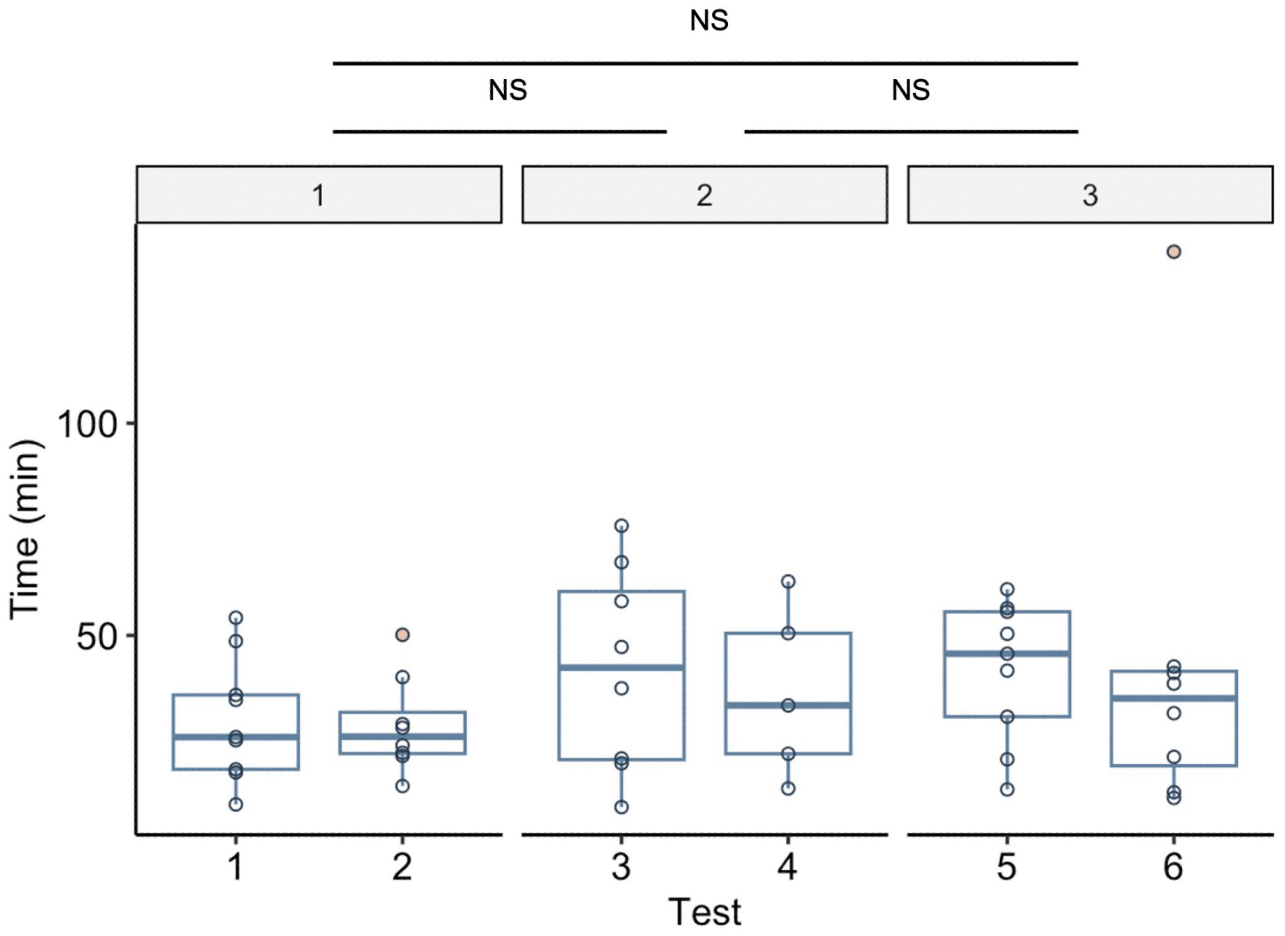
Latency until carrying (decision made) across test types and the number of differences. Neither test type nor attribute number significantly affected the latency. The middle thick line for each box shows the median, and the lower and upper edges show the first and third quartiles, respectively. The whiskers denote the maximum and minimum values that are not outliers. Circles represent the latency of each colony, and pink dots represent outliers, defined as data points that fall 1.5 interquartile ranges below the first quartile or above the third quartile.

Neither attribute number (χ^2^ = 5.852, df = 2, p = 0.054) nor test type (χ^2^= 8.482, df = 5, p = 0.132) affected the latency until carrying (Fig 4). Trial order significantly affected choice latency in both models in an ANOVA (model with attribute number: χ^2^ = 16.251, df = 1, p < 0.01; model with test type: χ^2^ = 19.702, df = 1, p < 0.01; S3 Fig). Colony size did not affect the latency significantly in either models, so it was removed from both models (attribute number: χ^2^ = 0.760, df = 1, p = 0.383; test type: χ^2^ = 0.659, df = 1, p = 0.417). There was also no effect of attribute number (χ^2^ = 2.625, df = 2, p = 0.269) or test type (χ^2^ = 5.022, df = 5, p = 0.413) on the latency until carrying was complete (S4 Fig).

### Probability of emigration

Neither attribute number (χ^2^ = 2.667, df = 2, p = 0.264) nor test type (χ^2^ = 4.260, df = 5, p = 0.513) influenced the probability of whether the colonies emigrated. Trial number did not influence this probability of emigration in either model (attribute number: χ^2^ = 0.325, df = 1, p = 0.569; test type: χ^2^ = 0.160, df = 1, p = 0.689) nor did colony size (attribute number: χ^2^ = 0.001, df = 1, p = 0.982; test type: χ^2^ = 0.015, df = 1, p = 0.902), so they were not included in the final models.

## Discussion

While multi-attribute decision problems are common across diverse taxa and decision-making contexts, the mechanisms by which animals make such complex decisions in many ecological settings remain unclear [4]. Here, we explored how the number (one, two, or three) and type (brightness, height, and width of entrance) of attributes differentiating potential nest sites influenced the *accuracy* and *latency* of nest-site selection in acorn ants (*Temnothorax curvispinosu*s). We found that the degree of difference between candidate nests affected the quality of the nest selected, but preferences for the more attractive nest were primarily explained by differences in brightness. We also found that the decision-making latency of colonies was similar across all choice contexts, regardless of attribute number or test type. Together, our results suggest that ants may be temporally constrained when choosing a nest site, and the consequences of poor decisions may change as nests differ in both attribute number and type.

Increasing the number of attributes differing between two potential nests led to more colonies selecting the target nests. These results may be explained in several non-mutually exclusive ways. The first is that increasing the differences between potential nests may help facilitate decisions for the more attractive nest, even when some attributes conflict. Various drift-diffusion models address how animals make decisions when confronted with multiple options (e.g., [5]). One such model is called the ‘tug-of-war model’ in which an individual comparatively evaluates two options, computing an index of relative attractiveness between them, which is used to rank both options [19,33]. The larger the difference between the two, the easier (and faster) a decision threshold is reached. Here, nests that varied in all three attributes may have facilitated this comparison, with more attributes creating greater contrast between two potential nests. Another explanation is that when nests vary slightly (i.e., only in one attribute), the fitness consequences of choosing an inferior option are small relative to the decision costs (regarding time or cognitive processing) [10,34]. This may be especially true in the current study, as nests that varied in one attribute either varied in cavity height or entrance size, both of which are less preferred attributes [14]. In either case, the fitness consequences of choosing the less-preferred nest are likely quite low.

Although attribute *number* influenced decisions between nest sites, attribute *type* appeared to be more important. When potential nests differed in only one attribute, cavity height or entrance width, ants did not select the nest we expected to be more attractive based on previous findings [14]. These two attributes appear to be generally less prioritized, although previous studies have shown that ants still show robust preferences when these attributes are manipulated in isolation [14,15]. Interestingly, preferences were detected in the opposite direction when height was manipulated in isolation: ants seemed to prefer the nest with the shorter cavity when height was the only attribute manipulated. These results may be explained by the possibility that by manipulating nest height, and keeping the width of the entrance the same, the overall area of the nest entrance was larger for the alternative nest in these tests. Another possible explanation is that we inadvertently reduced the internal nest space by manipulating cavity height and controlling for nest volume. In addition to the three attributes manipulated here, cavity volume may represent another attribute ants consider when selecting a potential nest site. Future studies could investigate whether and to what extent ant colonies consider this attribute when selecting potential nests.

Across all choice comparisons, we found that ants prioritized nest brightness over the cavity height or the entrance’s width. Such findings agree with previous work, which suggests that brightness is the most prioritized attribute ants attend to when selecting a new nest site [10,14,15,19]. In all test types where nest brightness was manipulated, colonies almost always chose the dark nest, regardless of other attribute differences. One explanation for this preference is that acorn ants are ecological engineers capable of transforming acorns and other nuts into suitable nesting habitats for their colonies. They can manipulate certain structural features of the nest, for instance, by excavating the interior to increase volume and modifying entrance holes to regulate access [35,36]. They can even create an additional entrance to an artificial cavity [36]. Other aspects of potential nest sites, like internal brightness, however, are fixed and cannot be altered once a nest is chosen [14]. As a result, acorn ants may place greater value on fixed attributes during nest selection, prioritizing them over attributes they can modify post-occupation. Although we did not have a manipulation where brightness varied independently, based on previous findings, we would expect ants to prefer the darker nest [10,14,15,19]. Future studies could exclude brightness as an attribute and test whether acorn ants adjust their valuation of fixed versus modifiable nest attributes depending on ecological context or colony state. For instance, one can experimentally manipulate the ease with which colonies can modify nest structures after occupation by fixing the cavity volume and allowing modification of the width of the entrance with debris provided.

In many decision-making systems, improvements in accuracy often come at the cost of slower decisions, reflecting a speed-accuracy tradeoff [20]. In our study, however, colonies increased in accuracy, but not latency, with increasing numbers of attributes differentiating potential nests (especially brightness), suggesting that ant colonies may be able to improve accuracy without incurring temporal costs in this context. Here, colonies spent, on average, around 68 minutes completing the emigration process, which is shorter than latencies found in previous experiments [7,10]. We also found an effect of trial number on latency, with colonies choosing faster across subsequent tests. Although we allowed colonies to remain in their home nest for 4–7 days between trials, these findings suggest that colonies became familiar with the emigration process across successive emigrations. We also observed colonies that failed to make a decision during the trial period. It is possible that these colonies would have eventually done so if given more time, thereby affecting our average decision time; however, we consider this unlikely, as these colonies were not sending out scouts and actively searching for a new nest. Differences between our findings and those of previous studies could possibly be explained by variation in the size of colonies and/or the distance between home nests and new nests. There could also be variation in the quorum thresholds and trips of tandem running of colonies, which was indirectly measured and may affect the time colonies spent exploring the options and making a decision. Future studies can explore whether similar variation in choice complexity leads to differences in the scouting or tandem running behavior and subsequent quorum thresholds.

One possible explanation for the short latencies found across choice contexts here is that colonies process multiple nest attributes in parallel through the distributed activity of scouts, allowing faster consensus formation [19]. Alternatively, colonies may rely on decision rules that prioritize salient attributes, such as brightness, enabling efficient evaluation [15]. Notably, the critical factor in nest-site selection here was not how different the nests were, but rather what specific differences existed between them. The absence of clear speed-accuracy tradeoffs in this study may also reflect ecological pressures favoring rapid emigration. Given the complexity of information processing required for decision-making, particularly in unpredictable environments, prioritizing rapid decisions over prolonged deliberation may be more advantageous for survival [37]. Inhabiting a poor nest sooner, which still provides temporary shelter and a chance to find a new one in the future, is likely a better alternative than risking predation or desiccation. This may lead colonies to maintain consistent decision speeds even when more information is available. Similar findings in cavity-nesting ants indicate that collective mechanisms, such as quorum thresholds and positive feedback, can buffer the expected trade-off between speed and accuracy, highlighting the efficiency of decentralized decision-making systems [21,38].

In the fourth test condition, all colonies chose the alternative nest, which was considered less ideal, being bright, short, and narrow. It is possible that these colonies preferred the alternative nest because of the wider cavity space, as previously mentioned. Notably, in this test condition, only half of the colonies emigrated. This may be due to the perceived difference between the previous home nest and the new candidate nests, as the home nest originally consisted of better attributes (dark, tall, with a narrow entrance) and previous work has shown that ants are sensitive to recent experience when selecting nests [15,21]. Another non-mutually exclusive possibility is that ants experienced choice overload, a phenomenon in which decision quality deteriorates with an increasing number of options. This typically occurs when decision makers are confronted with more options, but it may also occur as options vary along multiple attributes [39]. One of the symptoms of choice overload is choice deferral, where a decision is put off until a later time [39]. In humans, one driver of this phenomenon is assortment complexity, where available options are similar and no dominant option exists [39,40]. A previous study has explored choice overload in another context in house-hunting ants, and found that individual ants experience choice overload with an increase in the number of potential nests [41]. In their study, they found that when increasing the number of potential nests from two to eight, individual ants stopped selecting preferred nests and began choosing randomly [41]. Future studies in ants and other animals could help determine how both the number of options and the attributes characterizing them contribute to choice overload, and how this may differ at the individual and colony level.

Because colonies arrive at a decision through a recruitment system that generates positive feedback, decisions are guided by interactions among ants rather than any single individual [8]. Insect colonies show consistent preferences that are not simple summations of comparisons made by individual workers but instead emerge from interactions among individuals who might only have knowledge of one available option [19,41,42]. Previous studies have found notable differences between individual ants and colonies regarding the rationality of their decision-making. For instance, individuals are susceptible to choice overload and decoy effects, whereas colonies are not [18,41]. Individual ants also take longer to complete an emigration when choosing between two similar nests than between two dissimilar nests, whereas colonies take less time when nests are similar [10]. Finally, colonies outperformed individuals in a difficult perceptual task, but individuals performed better than groups when the task was easy [43]. Such findings suggest that in our study, evaluating nest sites according to all three attributes and weighing those attributes accordingly may be a straightforward task at a colony level. However, individual ants performing the same task may show different patterns. Additionally, while we did not directly quantify the repeatability of colony-level variation, some colonies seemed generally more active, with scouts promptly searching for new nests and recruiting other ants, while other colonies were less active, only sending one or two scouts during the emigration process. Future studies could explore whether colony-level variation in activity or overall efficiency influences decision-making behaviors.

Taken together, our findings suggest that the degree of differences does not affect the decision-making latency in house-hunting acorn ants, suggesting that ant colonies searching for a new nest might be constrained temporally when selecting a new nest site. However, differences influenced the accuracy of decisions about which nests to emigrate to, particularly when nest brightness was manipulated, indicating that increasing the number of attributes facilitates nest-site selection. When multiple attributes characterize nests, this may have important consequences for decision accuracy and latency in ants. Such results highlight the importance of incorporating more complexity into choice studies to understand how animals make decisions in the wild.

## Supporting information

S1 Table Nest attributes in previous studies. The table includes the dimensions of nests used in previous experiments. Pratt & Pierce (2001) used real acorns in the experiment. The values here are from the results after they measured the attributes of the acorns used.
(DOCX)

S2 Table Number of colonies that emigrated and did not emigrate. The table includes the number of colonies in each test that completed emigration and did not complete the process with the proportion of emigration.
(DOCX)

S1 Dataset Dataset used in data analysis. The data include all raw data collected in the experiment.
(CSV)

S1 File R codes. **R codes used in data analysis.** The R file contains the codes used for data analysis.
(R)

S1 Fig Combinations of materials used for nests. These are cross-sectional views of the nests. Brown indicates wood, and blue indicates glass. Dotted lines outline the shape of the cavity.
(TIF)

S2 Fig Accuracy across trials. Each line represents the accuracy of each colony across the trials it underwent. Choice 1 is the target, and choice 2 is the alternative. There is a significant effect of trial on choice accuracy in ANOVA but not in post hoc comparisons.
(TIF)

S3 Fig Latency until carrying across trials. Each line represents the latency change of each colony across trials it underwent. There is a significant effect of trial on choice latency in ANOVA but not in post hoc comparisons.
(TIF)

S4 Fig Latency until end of trial (decision finalized) across test types and the number of differences. Neither test type nor attribute number significantly affected the latency. Circles represent the latency of one colony, and pink dots represent outliers.
(TIF)
