# Supplementary material for: Nest attributes influence choice accuracy, but not decision latency in acorn ants
Source: PLoS One. 2026 Jan 16;21(1):e0329528. doi: 10.1371/journal.pone.0329528 (PMC12810839; doi:10.1371/journal.pone.0329528)
Supplement: S1 Table — The table includes the dimensions of nests used in previous experiments. Pratt & Pierce (2001) used real acorns in the experiment. The values here are from the results after they measured the attributes of the acorns used. (DOCX) [file pone.0329528.s001.docx]

**S1 Table. Nest attributes in previous studies.** The table includes the dimensions of nests used in previous experiments. Pratt & Pierce (2001) used real acorns in the experiment. The values here are from the results after they measured the attributes of the acorns used.

| Study | | Current | Mallon et al. (2001) | Franks et al. (2003) | Pratt & Pierce (2001) | Sasaki & Pratt (2013) | Sasaki et al. (2018) |
| --- | --- | --- | --- | --- | --- | --- | --- |
| Width of entrance | Wide | 4 mm | 23 mm | 4 mm | 4.4 mm^2^ | 5.5 mm | 5.5 mm |
|  | Medium | N/A | N/A | 2 mm | N/A | N/A | N/A |
|  | Narrow | 2 mm | 2 mm | 1 mm | 0.93 mm^2^ | 2 mm | 2 mm |
| Cavity height | Thick | 3 mm | 1.6 mm | 1.6 mm | 3.8 mm | N/A | N/A |
|  | Thin | 1.5 mm | 0.8 mm | 0.8 mm |  | N/A | N/A |
