# Supplementary material for: Nest attributes influence choice accuracy, but not decision latency in acorn ants
Source: PLoS One. 2026 Jan 16;21(1):e0329528. doi: 10.1371/journal.pone.0329528 (PMC12810839; doi:10.1371/journal.pone.0329528)
Supplement: S2 Table — The table includes the number of colonies in each test that completed emigration and did not complete the process with the proportion of emigration. (DOCX) [file pone.0329528.s002.docx]

**S2 Table. Number of colonies that emigrated and did not emigrate.** The table includes the number of colonies in each test that completed emigration and did not complete the process with the proportion of emigration.

| Test | No. of colonies emigrated | No. of colonies not emigrated | Proportion of emigration |
| --- | --- | --- | --- |
| 1 | 9 | 1 | 90% |
| 2 | 8 | 2 | 80% |
| 3 | 8 | 2 | 80% |
| 4 | 5 | 5 | 50% |
| 5 | 9 | 1 | 90% |
| 6 | 8 | 2 | 80% |
